# Rapid unimolecular reaction of stabilized Criegee intermediates and implications for atmospheric chemistry

**DOI:** 10.1038/s41467-019-09948-7

**Published:** 2019-05-01

**Authors:** Bo Long, Junwei Lucas Bao, Donald G. Truhlar

**Affiliations:** 1 School of Materials Science and Engineering, Guizhou Minzu University, 550025 Guiyang, China; 2 Department of Chemistry, Chemical Theory Center, and Supercomputing Institute, University of Minnesota, Minneapolis, MN 55455-0431 USA

**Keywords:** Atmospheric chemistry, Reaction kinetics and dynamics, Computational methods

## Abstract

Elucidating atmospheric oxidation mechanisms is necessary for estimating the lifetimes of atmospheric species and understanding secondary organic aerosol formation and atmospheric oxidation capacity. We report an unexpectedly fast mechanistic pathway for the unimolecular reactions of large stabilized Criegee intermediates, which involves the formation of bicyclic structures from large Criegee intermediates containing an aldehyde group. The barrier heights of the mechanistic pathways are unexpectedly low – about 2–3 kcal/mol – and are at least 10 kcal/mol lower than those of hydrogen shift processes in large *syn* Criegee intermediates; and the calculated rate constants show that the mechanistic pathways are 10^5^-10^9^ times faster than those of the corresponding hydrogen shift processes. The present findings indicate that analogous low-energy pathways can now also be expected in other large Criegee intermediates and that oxidative capacity of some Criegee intermediates is smaller than would be predicted by existing models.

## Abstract (web summary)

Criegee intermediates have received much attention in atmospheric chemistry because of their importance in ozonolysis mechanisms. Here, using quantum mechanical kinetics, the authors reveal an unexpectedly fast mechanistic pathway for unimolecular reactions of large stabilized Criegee intermediates.

## Introduction

Ozonolysis of alkenes is an important step in atmospheric mechanisms that control the oxidative capacity of the atmosphere and the production of sulfuric acid and secondary organic aerosols that are important in climate models^1–5^.

The first step in ozonolysis is 1,3-dipolar cycloaddition of O_3_ to the alkene to produce a primary ozonide (POZ), which is a compound containing an O–O–O group. The POZs produced by ozonolysis of acyclic or exocyclic alkenes decompose to produce a carbonyl oxide—called a Criegee intermediate (CI)—and an aldehyde or ketone. The reaction is exothermic, and as a consequence the CI is produced hot^6^, which is called chemical activation. Previous investigation has shown that hot *syn* CIs from cycloalkene ozonolysis mainly undergo a hydrogen shift reaction to produce a vinyl hydroperoxide^6^, which decomposes to produce a hydroxyl radical; this mechanism is called the prompt OH mechanism. But a fraction of the CIs are collisionally thermalized, producing what are called stabilized CIs or SCIs. The SCIs may also undergo hydrogen shift reactions, but their longer life allows other reactions to compete, especially unimolecular rearrangement to dioxiranes and bimolecular reactions with a variety of atmospheric constituents, such as water, water dimers, SO_2_, or other acids^6–13^.

The POZs produced by ozonolysis of endocyclic alkenes produce bifunctional molecules containing both a carbonyl oxide group and a carbonyl group. They may also react by a hydrogen shift, a unimolecular rearrangement to produce a dioxirane, or by a bimolecular reaction, but they also have other possibilities such as intramolecular attack of the carbonyl oxide on the carbonyl, producing a secondary ozonide (SOZ), which is a compound containing an O–C–O–O group.

**Fig. 1:**
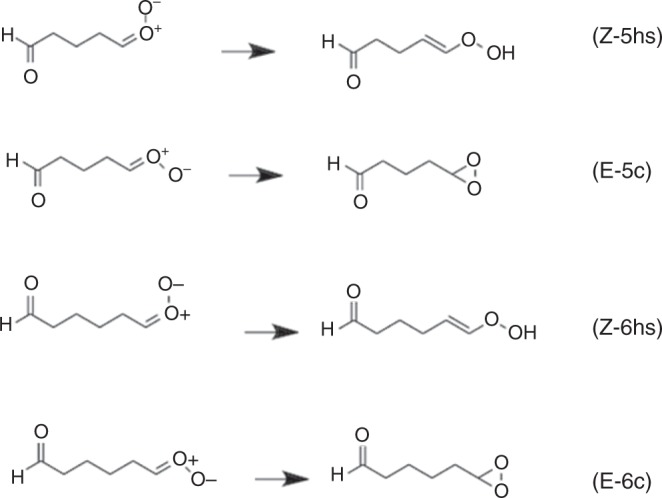
Hydrogen shift and dioxirane cyclization reactions

Direct detection of gaseous Criegee intermediates is mainly limited to small CIs^8–16^. Larger CIs capable of producing SOZs include those from ozonolysis of cyclic terpenes and terpenoids, which are volatile organic compounds with high biogenic concentrations in the atmosphere. Experimental data on the large Criegee intermediates are scarce. Here, we investigate the unimolecular reactions two large Criegee intermediates, namely C_5_H_8_O_3_ and C_6_H_10_O_3_, which have structural features that may be considered as prototypes for CIs produced by ozonolysis of cyclic terpenes. The hydrogen shift and dioxirane cyclization reactions are shown in Fig. 1, in which the reactions labeled Z are for *syn* species and those labeled E are for *anti*.

**Fig. 2:**
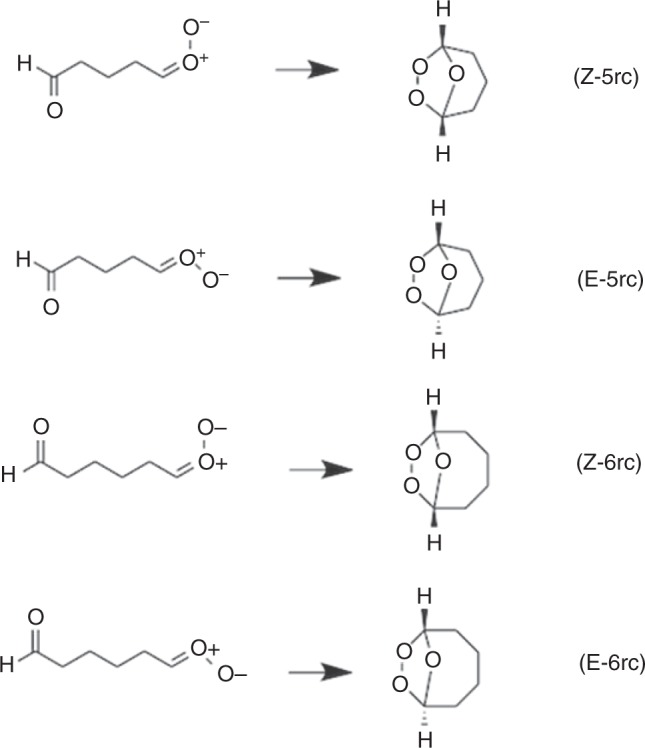
Bicyclic ring closure reactions

In the present paper, we study reactions of SCIs, and we report reactions that in the *syn* case are much faster than the above hydrogen shift reactions that are used in standard mechanisms. In particular, we report that the bicyclic ring closure reactions as shown in Fig. 2 are much faster than the hydrogen shift for the *syn* case and of comparable reaction rate to the dioxirane cyclization reaction for the *anti* case. We show here that these reactions are important when the Criegee intermediates have aldehyde functional groups, but they should probably also be important when they have other functional groups such as carboxylic acid functional groups. For example, monoterpenes^17^ such as limonene, 2-carene, 3-carene, phellandrene, terpinene, and terpinolene have cyclic structures whose ozonolyisis leads to the formation of CHO and HCOO groups. The Criegee intermediates resulting from ozonolysis of unsaturated oxygenated organic compounds^18^ such as unsaturated aldehydes^19^ and fatty acids^20^ may also contain other groups.

## Results

### General remarks

The importance of including multiple torsions and multiple transition states in reactions of large molecules has been emphasized previously^21,22^. In the present work we employ multistructural variational transition state theory^22^ to include all rotamer structures in our rate calculations; further details of the kinetics methods are given in the Methods section.

Although we use standard notation, it is worthwhile to review some definitions since some readers may not know them. The classical barrier height is the difference in Born–Oppenheimer potential energy between the conventional transition structure (saddle point) and the equilibrium reactant. When we give enthalpies of activation in this article, they are the values calculated by conventional transition state theory in which the transition state is located at the saddle point, and they are for 0 K; thus we label these quantities at $\Delta H_0^\ddagger$, and they are equal to the classical barrier height plus the zero-point energy of the transition structure minus the zero-point energy of the reactant. Activation energies are temperature-dependent and are defined in terms of Arrhenius plots as −*R*dln*k*/d(1/*T*), where *R* is the gas constant, *k* is the rate constant, and *T* is the temperature.

Large Criegee intermediates have many conformational structures, for example, *syn*-C_6_H_10_O has 112 structures and the transition state of E-6c-TS has 182 distinguishable structures. The numbers of structures for other species (reactants and transition states) are given in the Supplementary Information. Multiple structures are a form of anharmonicity since a harmonic potential leads to only a single structure. Our kinetics calculations include all distinguishable structures and the anharmonicity that they generate; we use the MS-T method^23^ to treat the multiple-structure anharmonicity, torsional-potential anharmonicity, and vibration–rotation coupling. The effect of these anharmonicities on the reaction rates is shown in Supplementary Table 6; over the 190–298 K temperature range, it varies from a factor of 0.025 to a factor of 0.74 for the bicyclic ring closure reactions, which corresponds to decreasing the rate by as much as a factor of 40 as compared to the quasiharmonic approximation. The effects on the rate are smaller—but not negligible—for the other kinds of reaction, varying between factors of 0.55 and 1.57 for the hydrogen shift reactions and between factors of 0.27 and 1.28 for dioxirane cyclization reactions. In the rest of the discussion, with one exception (Z-5hs-TS2, which is explained below), we discuss only the lowest-energy structure of each species.

### The unimolecular reactions of *syn*-C_5_H_8_O_3_

**Fig. 3:**
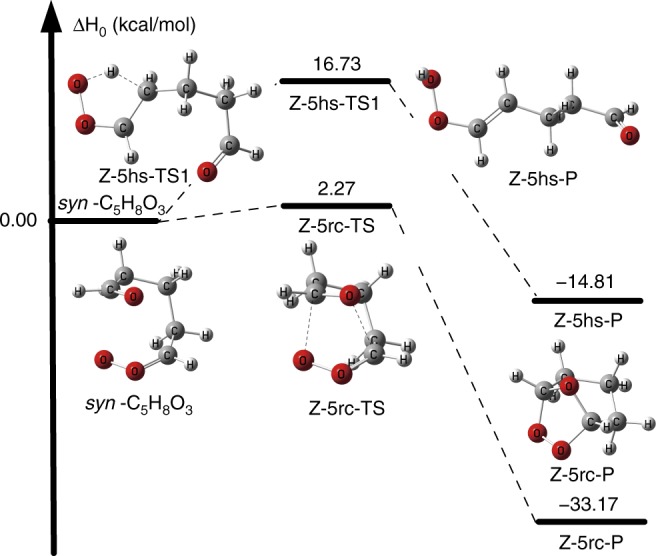
The calculated enthalpy profile of *syn*-C_5_H_8_O_3_

The bicyclic ring closure reaction we find for *syn*-C_5_H_8_O_3_ is labeled above as Z-5rc and is shown Fig. 3, which also shows the enthalpy profile at 0 K (i.e., the summation of electronic energy and the zero-point vibrational energy) computed by CCSD(T)-F12a/jun-cc-pVTZ based on MN15-L/MG3S geometries. The reaction is analogous to the bimolecular reaction of CH_2_OO with HCHO^24^. The previous assumption would have been that the dominant unimolecular reaction of this *syn*-Criegee intermediates would be the 1,4-hydrogen shift process that we have labeled Z-5hs, which is also shown in Fig. 3. The transition states for the hydrogen shift reactions have both an equatorial structure and an axial structure: Z-5hs-TS1 (shown in Fig. 3) and Z-5hs-TS2 (shown in Supplementary Fig. 1). The equatorial structure has a lower energy.

The bicyclic ring closure reaction involves the concerted formation of two bonds; it involves binding of the carbonyl carbon atom of the CHO group to the terminal oxygen atom of the HCOO group and binding of the carbonyl oxygen atom of the HCO group to the carbon atom of the HCOO group. This process is similar to the unimolecular reactions of other Criegee intermediates in the ozonolysis of isoprene and β-myrcene^25–27^, where the HCOO group attacks the C=C double bond. However, the enthalpies of activation at 0 K are above 10 kcal/mol for the similar unimolecular reactions of the HCOO group with the C=C group;^25–29^ this is also qualitatively consistent with the corresponding CH_2_OO+HCHO and CH_2_OO+C_2_H_4_ reactions, where the enthalpy of activation at 0 K for the CH_2_OO+C_2_H_4_ reaction is higher than that of the CH_2_OO+HCHO reaction^24,30^. There are two different transition state structures for SOZ formation in *syn*-C_5_H_8_O_3_ (see Supplementary Fig. 2). Here, we only consider the lowest transition state Z-5rc-TS because Z-5rc-TS is 3.37 kcal/mol lower than Z-5rc-TS1 (see Supplementary Fig. 2). The two C–O bond distances of the new bonds at the transition state (Z-5rc-TS) are respectively 2.11 and 2.15 Å, which may be compared to values of 1.45 and 1.42 Å in the product.

Table 1 shows that the enthalpy of activation (all enthalpies of reaction and enthalpies of activation in the text are given for 0 K to illustrate the energetics in the well-defined low-temperature limit, but the results are similar at atmospheric temperatures;  activation energies at atmospheric temperatures are given in the SI) for the SOZ-forming bicyclic ring closure as calculated by CCSD(T)-F12a/jun-cc-pVTZ//MN15-L/MG3S is 2.27 kcal/mol (see Table 1) and that this is lower than that of the hydrogen shift process by about 14.5 kcal/mol (i.e., 16.73−2.27). Moreover, Fig. 3 shows that the product Z-5rc-P of the bicyclic ring closure reaction is much more stable than the product Z-5hs-P1 of the hydrogen shift reaction because the enthalpy of reaction for Z-5rc is 18.4 kcal/mol lower (i.e., 33.17−14.81). The present finding is different from the previously reported results which indicated a low barrier for SOZ formation only for the case of six carbons in the product ring^31,44^.

**Table 1:** The enthalpies of activation (kcal/mol) at 0 K of the unimolecular reactions

Method					
	Z-5rc-TS	Z-5hs-TS1	Z-5hs-TS2	E-5rc-TS	E-5c-TS
Post-CCSD(T) approximation					
WMS^a^	2.47	16.55	21.51	14.13	13.97
Approximations to CCSD(T)/CBS					
W2X^a^	2.45	16.46	21.31	14.15	13.82
CCSD(T)-F12a/jun-cc-pVTZ^a^	2.27	16.73	21.33	13.74	13.73
Methods affordable for direct dynamics					
MN15-L/MG3S	2.83	18.28	21.90	13.19	14.95
MN15-L/maug-cc-pVTZ	2.33	17.49	21.33	12.83	14.60

	Z-6rc-TS	Z-6hs-TS1	Z-6hs-TS2	E-6rc-TS	E-6c-TS
WMS^a^	2.05	16.13	18.52	5.11	14.72
CCSD(T)-F12a/jun-cc-pVTZ^a^	1.47	15.83	18.33	4.82	14.36
MN15-L/MG3S	2.73	16.54	19.54	5.70	14.35
MN15-L/maug-cc-pVTZ	2.72	16.03	18.93	5.33	14.18

^a^Geometry optimization and frequency calculation at the MN15-L/MG3S level followed by a single-point energy calculation with the indicated method

To further check the reliability of the CCSD(T) enthalpy of activation, we performed two higher-order calculations, and the results of these are shown in Table 1. The quite different very-high-level W2X^32^ and WMS^33^ methods almost give identical results for Z-5rc; this shows it is not required to carry out post-CCSD(T) calculations on the bicyclic ring closure reaction of the large-sized Criegee intermediate *syn*-C_5_H_8_O_3_, and the good agreement of activation enthalpies in Table 1 shows that we can trust the CCSD(T) calculation for further studies. A further indication that post-CCSD(T) theory is not needed is that the [*E*(CCSDT(Q))−*E*(CCSD(T))] contribution to the Z-5rc barrier height calculated with the VDZ(d)^32^ basis is only 0.07 kcal/mol (Supplementary Table 1).

The WMS, W2X, and CCSD(T) calculations in Table 1 are too expensive for direct dynamics calculations. We selected the MN15-L density functional for the present work because of its good performance demonstrated previously for strongly correlated systems and barrier heights^34^ and based on its successful use in our previous investigations of small Criegee intermediates^35,36^. The results in Table 1 then provide further confirmation of its usefulness for the present problem. In particular, the MN15-L/MG3S and MN15-L/maug-cc-pVTZ density functional calculations provide enthalpies of activation of respectively 2.8 and 2.3 kcal/mol for Z-5rc, in good agreement with the high-level WMS value of 2.5 kcal/mol; this shows more specifically that MN15-L/MG3S can provide useful results for describing the bicyclic ring closure reactions of Criegee intermediates. This is important because MN15-L is less expensive and can be used for full rate constant calculations. Thus, MN15-L/MG3S is selected for the direct dynamics components of our rate constant calculations.

### The unimolecular reactions of *anti*-C_5_H_8_O_3_

The enthalpy of activation for isomerization of *anti*-C_5_H_8_O_3_ to *syn*-C_5_H_8_O_3_ is 35.43 kcal/mol as shown in Supplementary Fig. 3. The isomerization mechanism between *anti*-C_5_H_8_O_3_ and *syn*-C_5_H_8_O_3_ is expected to be similar to that in small-sized Criegee intermediates^28,35,37^. Thus, *syn*-C_5_H_8_O_3_ and *anti*-C_5_H_8_O_3_ act as independent reactants.

**Fig. 4:**
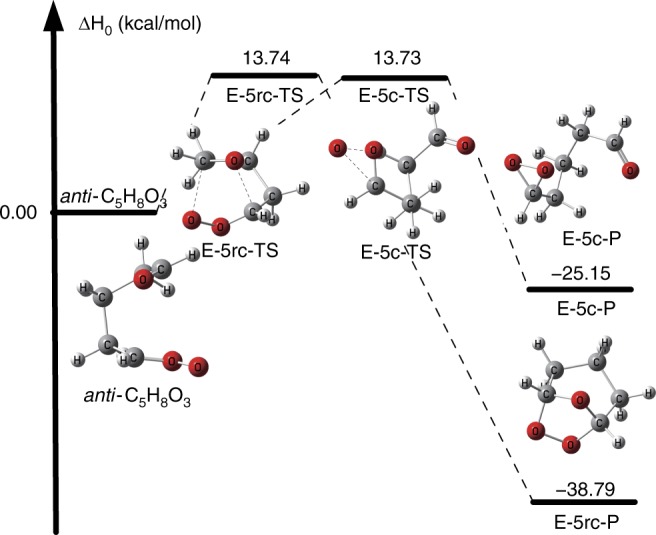
The calculated enthalpy profile of *anti*-C_5_H_8_O_3_ by CCSD(T)-F12a/jun-cc-pVTZ

A bicyclic ring closure reaction is also found for *anti*-C_5_H_8_O_3_, as seen in Fig. 4 (which also shows the enthalpy profile at 0 K computed by CCSD(T)-F12a/jun-cc-pVTZ//MN15-L/MG3S); however, it has a much higher barrier in this case. Furthermore, there is only one transition state structure for SOZ formation in *anti*-C_5_H_8_O_3_. Table 1 shows that that the enthalpies of activation for E-5rc and E-5c are both calculated to be 13.7 kcal/mol; this indicates that the E-5rc reaction, although not dominant, should not be neglected in computing the removal rate of *anti*-C_5_H_8_O_3_.

### The unimolecular reactions of *syn*-C_6_H_10_O_3_ and *anti*-C_6_H_10_O_3_

We also investigate the *syn*- and *anti*-Criegee intermediates from the ozonolysis of cyclic C_6_H_10_. Supplementary Figure 4 shows that the dominant reaction pathway of *syn*-C_6_H_10_O_3_ is similar to the unimolecular reaction of *syn*-C_5_H_8_O_3_. The activation enthalpy for Z-6rc is calculated to be 1.5 kcal/mol by CCSD(T)-F12a/jun-cc-pVTZ//MN15-L/MG3S, which is about 14.4 kcal/mol lower than that of the previously suggested^38^ hydrogen transfer shift Z-6hs-TS1 in Table 1; this means that this pathway is much faster than that of the hydrogen shift. However, the previous theoretical results reported a much higher barrier of 5.1 (ref. ^6^) or 8.5 kcal/mol^39^ for the bicyclic ring closure reactions of *syn*-C_6_H_8_O_3_. A key issue in this discrepancy is that one must find the lowest-energy transition state, and there are four different transition state structures for SOZ formation from *syn*-C_6_H_10_O_3_ (see Supplementary Fig. 5) and two different transition state structures for the bicyclic ring closure reaction in *syn*-C_5_H_8_O_3_; this situation occurs because each of the two central CH_2_ groups in *syn*-C_6_H_10_O_3_ has two different pathways leading to SOZ formation; in comparison, the one central CH_2_ group in *syn*-C_5_H_8_O_3_ leads to two pathways. This example illustrates how there are more transition state structures for SOZ formation and how one locates more transition state structures when the carbon chain becomes longer. The lowest-energy transition state Z-6rc-TS is considered here because the CCSD(T)-F12a/jun-cc-pVTZ//MN15-L/MG3S calculations indicate that it is 1.97 kcal/mol lower than the second lowest structure, 3.89 kcal/mol lower than the third lowest structure, and 7.68 kcal/mol lower than the fourth lowest structure (see Supplementary Fig. 5).

The enthalpy of activation for isomerization of *syn*-C_6_H_10_O_3_ to *anti*-C_6_H_10_O_3_ is 37.37 kcal/mol (see Supplementary Fig. 6); this shows that *syn*-C_6_H_10_O_3_ and *anti*-C_6_H_10_O_3_ can be considered to be independent reactants.

For *anti*-C_6_H_10_O_3_, there are two different transition state structures for SOZ formation (see Supplementary Fig. 7). We consider E-6rc-TS because E-6rc-TS is 3.41 kcal/mol lower than E-6rc-TS1 (see Supplementary Fig. 7). Table 1 and Supplementary Fig. 8 show an enthalpy of activation of 4.8 kcal/mol for bicyclic ring closure E-6rc, which is 9.5 kcal/mol lower than that of the dioxirane cyclization process E-6c-TS. The enthalpy of activation at 0 K for E-6rc is about 9 kcal/mol lower than that of E-5rc because of different chain strain between C_5_ and C_6_ Criegee intermediates. Thus, for the C_6_ case, this reaction dominates the previously known reactions for both *syn* and *anti*.

Cyclic C_6_H_10_ represents the skeleton of monoterpenes such as limonene, 2-carene, 3-carene, phellandrene, terpinene, and terpinolene^17^. Therefore, the present conclusions are expected to be important for the monoterpenes; however, the SOZ-forming bicyclic ring closure was previously only included in the atmospheric modeling of the ozonolysis of β-caryophyllene^40^.

### Kinetics

The calculated rate constants are given in Table 2 and Supplementary Table 3. The unimolecular rate constants were fitted using the following formula:

1 $$k = A\left( {\frac{{T + T_0}}{{300}}} \right)^n{\mathrm{exp}}\left[ { - \frac{{{\mathrm{E}}(T + T_0)}}{{R(T^2 + T_0^2)}}} \right].$$

**Table 2:** The calculated rate constants (s^-1^) of the unimolecular reactions

	200 K	220 K	240 K	260 K	280 K	298 K
Z-5rc	1.8 × 10^9^	2.7 × 10^9^	3.9 × 10^9^	5.1 × 10^9^	6.3 × 10^9^	7.1 × 10^9^
Z-5hs	5.2 × 10^0^	1.7 × 10^1^	5.7 × 10^1^	1.9 × 10^2^	6.2 × 10^2^	1.7 × 10^3^
E-5rc	1.8 × 10^−5^	3.9 × 10^−4^	4.8 × 10^−3^	3.9 × 10^−2^	2.3 × 10^−1^	9.0 × 10^−1^
E-5c	1.6 × 10^−3^	4.0 × 10^−2^	5.8 × 10^−1^	5.7 × 10^0^	4.1 × 10^1^	1.9 × 10^2^
Z-6rc	2.4 × 10^8^	2.8 × 10^8^	3.0 × 10^8^	3.2 × 10^8^	3.2 × 10^8^	3.2 × 10^8^
Z-6hs	9.0 × 10^0^	2.2 × 10^1^	5.5 × 10^1^	1.4 × 10^2^	3.4 × 10^2^	7.9 × 10^2^
E-6rc	1.4 × 10^6^	3.6 × 10^6^	7.6 × 10^6^	1.3 × 10^7^	1.9 × 10^7^	2.4 × 10^7^
E-6c	7.4 × 10^−4^	2.0 × 10^−2^	3.0 × 10^−1^	2.8 × 10^0^	1.8 × 10^1^	7.8 × 10^1^

The fitted parameters are provided in Supplementary Table 4. Table 2 shows that the SOZ-forming bicyclic ring closures Z-5rc and Z-6rc of *syn*-C_5_H_8_O_3_ and C_6_H_10_O_3_ are about 10^5^–10^9^ times faster than the previously suggested hydrogen transfer process (Z-5hs and Z-6hs) and the SOZ-forming bicyclic ring closure E-6rc is about 10^6^–10^8^ times faster than the previously suggested dioxirane cyclization reaction.

Supplementary Table 5 gives the temperature-dependent activation energies *E*_a_ (computed as local slopes of Arrhenius plots^41^). Over the atmospherically relevant temperature range 190–298 K, the activation energies for the ring closure reactions increase as the temperature is lowered; for example, *E*_a_ for Z-5rc increases from 1.1 to 2.0 kcal/mol.

In Table 2, the rate constants of Z-5rc are much faster than those of Z-6rc, while the enthalpies of activation for Z-5rc at 0 K is 0.42 kcal/mol higher than that of Z-6rc calculated by WMS (Table 1); this is mainly caused by the torsional anharmonicity (see Supplementary Table 6). For example, torsional anharmonicity decreases the rate constant at 298 K by a factor of 2 for Z-5rc and by a factor of 40 for Z-6rc. Therefore, the torsional anharmonicity can play important roles in determining the kinetics of bicyclic ring closure reactions of Criegee intermediates, and this conclusion extends to large systems as well as the present systems.

### Implications for atmospheric chemistry

Although SOZ formation is well known in the literature^6,31,38–40,42–44^ and previous investigation indicated that SOZ formation dominates over the hydrogen shift for the *syn* SCI from cycloalkenes ozonlysis^6^, the rates of reaction of large SCIs are unclear in previous work because of the different barrier heights for SOZ formation reported in the literature for the same Criegee intermediate. In particular, the barrier height was reported to be 5.1 (ref. ^6^) or 8.5 (ref. ^39^) kcal/mol for secondary ozonide formation in the *syn*-Criegee intermediate from cyclohexene ozonolysis. The primary objective of the present article was to provide data on the reaction rates for producing SOZs in gas-phase reactions of SCIs. The present calculations are for collisonally stabilized reactants, and we note that pressure-dependent kinetics calculations not reported here would be required to to fully understand and predict the extent of collisional stabilization of these large Criegee intermediates; future work to study this could be carried out using master equation simulations^45^ or the system-specific quantum Rice–Ramsperger–Kassel model^46^.

Unimolecular reactions of small Criegee intermediates, such as CH_2_OO^35,47–49^ *syn* and *anti*-CH_3_CHOO^35,37,50,51^ (CH_3_)_2_COO^36,52^ and *syn*/*anti*-C_2_H_5_CHOO^28,29^, were previously investigated by using both theoretical and experimental methods. The generally accepted view from these studies is that *syn-*Criegee intermediates react mainly by hydrogen shift (hs) reactions. Hydrogen shift reactions of Criegee intermediates from a methyl or ethyl group to the terminal oxygen atom are especially important because they lead to the formation of OH radical^11,36,50,51,53^ and can be the initial step for the formation of highly oxygenated molecules, which make a significant contribution to the formation of atmospheric organic aerosols^54,55^. The dominant pathway of small *anti*-Criegee intermediates is usually assumed to be cyclization (c) to a dioxirane^.^^35,37^ The enthalpies of activation of the unimolecular reactions of small Criegee intermediates are in the range between 15 and 30 kcal/mol^35–37^; this implies that the unimolecular reactions of small Criegee intermediates such as CH_2_OO and *anti*-CH_3_CHOO cannot compete well with bimolecular reaction with water, water dimer, SO_2_, or atmospheric acids^10,35,56^.

The bicyclic ring closure reactions that we report here are important for understanding the atmospheric lifetimes of large Criegee intermediates—not only for those from the ozonolysis of C_5_H_8_ and C_6_H_10_ but also for a wider class of compounds. While previous experimental and theoretical results have suggested that secondary ozonides (SOZ) are formed by unimolecular reactions in the ozonolysis of cycloalkenes^6,40^, the dominant pathways are generally assumed to be the same as those for the small Criegee intermediates. For example, recent studies of the ozonolysis of limonene, α-pinene, other monoterpenes, and indene included the hydrogen shift reaction as the only unimolecular step in the mechanisms^57–59^. Since the structural features of large Criegee intermediates can be quite different from those of small Criegee intermediates, it is important to understand whether they react by different mechanisms.

Previous investigations have shown that the unimolecular reactions of the simple Criegee intermediates occurring via hydrogen shifts lead to the formation of OH radical via the decomposition of -OOH groups^11,50,51,53^. However, our calculated results show that the unimolecular reactions of larger *syn*-Criegee intermediates from the ozonolysis of C_5_H_8_ and C_6_H_10_ predominantly undergo reactions in which no −OOH group is formed. Thus, the reactions limit OH production in the ozonolysis of C_5_H_8_ and C_6_H_10_; this further leads to the reduction of the oxidation capacity of the corresponding Criegee intermediates from cyclic C_6_ structures.

The reaction in three of the Criegee intermediates (*syn*-C_5_H_8_O_3_, *syn*/*anti*-C_6_H_10_O_3_) is so fast that the atmospheric lifetimes of three Criegee intermediates are much less than 10^−5^ s. However, the atmospheric lifetimes associated with the corresponding bimolecular reactions such as reactions with SO_2_, H_2_O, water dimer, and other atmospheric acids are often more than 10^−3^ s^56^. We conclude that large Criegee intermediates that are produced by ozonolysis do not act as potential oxidants to oxidize SO_2_ to SO_3_. This is especially important for monoterpenes with structural features similar to C_6_H_10._

The formation of highly oxidized molecules and radicals in the ozonolysis of cyclic monoterpenes plays a significant role in atmospheric organic aerosol formation^55^. Experimental results have shown that highly oxidized molecules and radicals can be formed in the ozonolysis of cyclohexene and limonene^38,57^. However, our results presented here clearly show that the dominant sink of *syn*- and *anti*-Criegee intermediates from six-carbon-atom cyclic monoterpenes is not the hydrogen shift mechanism as suggested in the literature; ^38,57^ Thus, the present investigation upsets one aspect of conventional thinking about the formation of highly oxidized molecules and radicals.

The reactions studied here are important in illustrating a more general phenomenon, namely that large Criegee intermediates may have lower energy unimolecular pathways that are analogous to the known bimolecular reactions of Criegee intermediates. For example, the ozonolysis of fatty acids^20^ can lead to Criegee intermediates with an COOH group, and these could react by a mechanism similar to the bimolecular reaction of Criegee intermediates with atmospheric acids with barrierless reactions^60,61^. Thus, the present results stimulate one to consider faster unimolecular reaction mechanisms of large Criegee intermediates.

### Summary

In the unimolecular reactions of Criegee intermediates, the accepted reaction mechanisms are hydrogen shifts and dioxirane cyclization. Here, we used electronic structure calculations and multistructural variational transition state theory with small-curvature tunneling to explore unimolecular reactions of large-sized stabilized Criegee intermediates produced in the ozonolysis of cyclic monoterpenes. We find that multistructural effects are important for determining the barrier heights and torsion anharmonicity.

For large *syn*-Criegee intermediates from the ozonolysis of cyclic monoterpenes, we find a mechanism involving unimolecular reactions with enthalpies of activation about 2–3 kcal/mol. We find that the unimolecular rate constants of the processes are 10^6^–10^9^ s^−1^ in the temperature range between 190 and 298 K. We find for *syn-*Criegee intermediates that a low-energy path for internal SOZ formation is available even with only five carbons in the product ring, whereas previous work^44^ incorrectly indicated that a minimum of six carbons is required.

We conclude that unexpectedly fast unimolecular reactions of Criegee intermediates significantly limit the oxidation capacity of Criegee intermediates, and we suggest that the reaction types with low-energy barriers as reported here should be widespread.

## Methods

### Computation of rate constants

Rate constants (*k*) were calculated by a dual-level approximation:

2 $$k = \frac{{\exp \left[ { - V_{{\mathrm{L}}2}^\ddagger /RT} \right]}}{{\exp \left[ { - V_{{\mathrm{L}}1}^\ddagger /RT} \right]}}k_{{\mathrm{L1}}}^{{\mathrm{MS - CVT/SCT}}}(T),$$

where level 1 (L1) of electronic structure employs the MN15-L local meta exchange-correlation functional^34^ with the MG3S basis set^62^, level 2 (L2) employs WMS, $V^\ddagger$ is the classical barrier height, *R* is the gas constant, *T* is the temperature, and MS-CVT/SCT is multistructural^63^ canonical variational theory^64,65^ with small-curvature tunneling^66^.

The conformational structures of the reactants and transition states were calculated by multistructural method with coupled torsional-potential anharmonicity (MS-T)^23^. The frequencies were scaled by the factor of 0.973 for MN15-L/MG3S as obtained in a standard way^67^ to account for vibrational anharmonicity and systematic errors in the electronic structure.

### Electronic structure methods

Our previous investigations have shown that the MN15-L functional can provide useful accuracy for the unimolecular reactions of small and medium-sized Criegee intermediates^35,36^. Our previous work also showed that obtaining quantitative rate constants for the unimolecular reactions of Criegee intermediates requires calculations beyond CCSD(T)^35,36^, but here we employ very-high-level calculations to show that CCSD(T) is sufficient for the low-energy pathways identified in this work. In addition, the composite methods WMS and W2X were used to do single-point calculations to show the reliability of the method chosen for direct dynamics. Even without the dynamics calculations, these high-level calculations of barrier heights would already show the importance of the bicyclic ring closure reactions.

It is impossible to put quantitative error bars on the electronic structure and rate constant calculations, but some discussion of reliability may be useful. Based on previous work, the uncertainties in the rate constant calculations are expected to be dominated by the electronic structure calculations rather than the errors in the dynamical parts of the calculations^68^. In electronic structure calculations, there are three factors to be considered: optimized geometries, vibrational frequencies, and single-point electronic energies^69^. In a previous paper, we have shown good agreement of CCSD(T) and MN15-L for optimized geometries and zero-point vibrational energies on similar systems^35^. Based on these tests and the previous work showing MN15-L is able to yield reasonably accurate vibrational frequencies^34^, the error of the electronic structure calculations is most likely not dominated by geometry errors or frequency errors. Previous experience with the methods used here indicates that the single-point electronic energies have typical errors of 1 kcal/mol or less^32,34,70^; this would cause errors in 300 K rate constants of a factor of 5 or less. This is consistent with previous work where we found that MN15-L yielded rate constants only a factor of 2 different from experimental results for the hydrogen shift of *syn*-CH_3_CHOO^35^, and it provided results consistent with experimental data in the hydrogen shift of (CH_3_)_2_COO^36^

## Supplementary information

Supplementary Information

Description of Additional Supplementary Files

Supplementary Data 1

## Data Availability

All data analyzed during this study are included in this published article and its supplementary information files. Supplementary information is available in the online version of the paper. Reprints and permissions information are available online at www.nature.com/reprints. Correspondence and requests for materials should be addressed to B.L., J.L.B., and D.G.T.

## Code Availability

The calculations were carried out using the *Polyrate*, *Gaussrate*, and *MSTor* computer codes that are available at no cost at http://truhlar.chem.umn.edu/content/software

## References

[1] Criegee R (1975). Mechanism of ozonolysis. Angew. Chem. Int. Ed. Engl..

[2] Johnson D, Marston G (2008). The gas-phase ozonolysis of unsaturated volatile organic compounds in the troposphere. Chem. Soc. Rev..

[3] Mauldin III, R. L. et al. A new atmospherically relevant oxidant of sulphur dioxide. *Nature* **488**, 193–196 (2012). 10.1038/nature11278 22874964

[4] Osborn DL, Taatjes CA (2015). The physical chemistry of Criegee intermediates in the gas phase. Int. Rev. Phys. Chem..

[5] Zhong J, Kumar M, Francisco JS, Zeng XC (2018). Insight into chemistry on cloud/aerosol water surfaces. Acc. Chem. Res..

[6] Chuong B, Zhang J, Donahue NM (2004). Cycloalkene ozonolysis: collisionally mediated mechanistic branching. J. Am. Chem. Soc..

[7] Vereecken L, Glowacki DR, Pilling MJ (2015). Theoretical chemical kinetics in tropospheric chemistry: methodologies and applications. Chem. Rev..

[8] Lee YP (2015). Perspective: Spectroscopy and kinetics of small gaseous Criegee intermediates. J. Chem. Phys..

[9] Su YT (2014). Extremely rapid self-reaction of the simplest Criegee intermediate CH_2_OO and its implications in atmospheric chemistry. Nat. Chem..

[10] Chao W, Hsieh JT, Chang CH, Lin JJM (2015). Direct kinetic measurement of the reaction of the simplest Criegee intermediate with water vapor. Science.

[11] Liu F, Beames JM, Petit AS, McCoy AB, Lester MI (2014). Infrared-driven unimolecular reaction of CH_3_CHOO Criegee intermediates to OH radical products. Science.

[12] Taatjes CA (2013). Direct measurements of conformer-dependent reactivity of the Criegee intermediate CH_3_CHOO. Science.

[13] Su YT, Huang YH, Witek HA, Lee YP (2013). Infrared absorption spectrum of the simplest Criegee intermediate CH_2_OO. Science.

[14] Taatjes CA (2008). Direct observation of the gas-phase Criegee intermediate (CH_2_OO). J. Am. Chem. Soc..

[15] Beames JM, Liu F, Lu L, Lester MI (2012). Ultraviolet spectrum and photochemistry of the simplest Criegee intermediate CH2OO. J. Am. Chem. Soc..

[16] Jr-Min Lin J, Chao W (2017). Structure-dependent reactivity of Criegee intermediates studied with spectroscopic methods. Chem. Soc. Rev..

[17] Atkinson R, Arey J (2003). Gas-phase tropospheric chemistry of biogenic volatile organic compounds: a review. Atmos. Environ..

[18] Mellouki A, Le Bras G, Sidebottom H (2003). Kinetics and mechanisms of the oxidation of oxygenated organic compounds in the gas phase. Chem. Rev..

[19] Rossignol S (2016). Atmospheric photochemistry at a fatty acid–coated air-water interface. Science.

[20] Kawamura K, Gagosian RB (1987). Implications of ω-oxocarboxylic acids in the remote marine atmosphere for photo-oxidation of unsaturated fatty acids. Nature.

[21] Vereecken L, Peeters J (2003). The 1,5-H-shift in 1-butoxy: a case study in the rigorous implementation of transition state theory for a multirotamer system. J. Chem. Phys..

[22] Yu T, Zheng J, Truhlar DG (2011). Multi-structural variational transition state theory. Kinetics of the 1,4-hydrogen shift isomerization of the pentyl radical with torsional anharmonicity. Chem. Sci..

[23] Zheng J, Truhlar DG (2013). Quantum thermochemistry: multistructural method with torsional anharmonicity based on a coupled torsional potential. J. Chem. Theory Comput..

[24] Aplincourt P, Ruiz-López MF (2000). Theoretical investigation of reaction mechanisms for carboxylic acid formation in the atmosphere. J. Am. Chem. Soc..

[25] Kuwata KT, Valin LC, Converse AD (2005). Quantum chemical and master equation studies of the methyl vinyl carbonyl oxides formed in isoprene ozonolysis. J. Phys. Chem. A.

[26] Kuwata KT, Valin LC (2008). Quantum chemical and RRKM/master equation studies of isoprene ozonolysis: methacrolein and methacrolein oxide. Chem. Phys. Lett..

[27] Deng P, Wang L, Wang L (2018). Mechanism of gas-phase ozonolysis of β-myrcene in the atmosphere. J. Phys. Chem. A.

[28] Yin C, Takahashi K (2017). How does substitution affect the unimolecular reaction rates of Criegee intermediates?. Phys. Chem. Chem. Phys..

[29] Vereecken L, Novelli A, Taraborrelli D (2017). Unimolecular decay strongly limits the atmospheric impact of Criegee intermediates. Phys. Chem. Chem. Phys..

[30] Vereecken L, Harder H, Novelli A (2014). The reactions of Criegee intermediates with alkenes, ozone, and carbonyl oxides. Phys. Chem. Chem. Phys..

[31] Kuwata KT, Kujala BJ, Morrow ZW, Tonc E (2011). Quantum chemical and RRKM/master equation studies of cyclopropene ozonolysis. Comput. Theor. Chem..

[32] Chan B, Radom L (2015). W2X and W3X-L: cost-effective approximations to W2 and W4 with kJ mol^–1^ accuracy. J. Chem. Theory Comput..

[33] Zhao Y (2018). Extrapolation of high-order correlation energies: the WMS model. Phys. Chem. Chem. Phys..

[34] Yu HS, He X, Truhlar DG (2016). MN15-L: a new local exchange-correlation functional for Kohn–Sham density functional theory with broad accuracy for atoms. Mol. solids J. Chem. Theory Comput..

[35] Long B, Bao JL, Truhlar DG (2016). Atmospheric chemistry of Criegee intermediates: unimolecular reactions and reactions with water. J. Am. Chem. Soc..

[36] Long B, Bao JL, Truhlar DG (2018). Unimolecular reaction of acetone oxide and its reaction with water in the atmosphere. Proc. Natl. Acad. Sci. USA.

[37] Kuwata KT, Hermes MR, Carlson MJ, Zogg CK (2010). Computational studies of the isomerization and hydration reactions of acetaldehyde oxide and methyl vinyl carbonyl oxide. J. Phys. Chem. A.

[38] Newland MJ (2018). The atmospheric impacts of monoterpene ozonolysis on global stabilised Criegee intermediate budgets and SO_2_ oxidation: experiment, theory and modelling. Atmos. Chem. Phys..

[39] Monge-Palacios M, Rissanen MP, Wang Z, Sarathy SM (2018). Theoretical kinetic study of the formic acid catalyzed Criegee intermediate isomerization: multistructural anharmonicity and atmospheric implications. Phys. Chem. Chem. Phys..

[40] Khan MAH (2017). A modeling study of secondary organic aerosol formation from sesquiterpenes using the STOCHEM global chemistry and transport model. J. Geophys. Res. Atmos..

[41] Truhlar DG, Gray JC (1978). Interpretation and emperature dependence of the energy of activation for the reactions H+Cl_2_, H_2_ + I, H + H_2_, and isotopic analogs. Chem. Phys. Lett..

[42] Hatakeyama S, Kobayashi H, Akimoto H (1984). Gas-phase oxidation of sulfur dioxide in the ozone-olefin reactions. J. Phys. Chem..

[43] Nguyen TL (2009). The gas-phase ozonolysis of β-caryophyllene (C_15_H_24_). Part II: a theoretical study. Phys. Chem. Chem. Phys..

[44] Vereecken L, Francisco JS (2012). Theoretical studies of atmospheric reaction mechanisms in the troposphere. Chem. Soc. Rev..

[45] Glowacki DR, Liang CH, Morley C, Pilling MJ, Robertson SH (2012). MESMER: an open-source master equation solver for multi-energy well reactions. J. Phys. Chem. A.

[46] Bao JL, Zheng J, Truhlar DG (2016). Kinetics of hydrogen radical reactions with toluene including chemical activation theory employing system-specific quantum RRK theory calibrated by variational transition state theory. J. Am. Chem. Soc..

[47] Berndt T (2015). Kinetics of the unimolecular reaction of CH_2_OO and the bimolecular reactions with the water monomer, acetaldehyde and acetone under atmospheric conditions. Phys. Chem. Chem. Phys..

[48] Chhantyal-Pun R, Davey A, Shallcross DE, Percival CJ, Orr-Ewing AJ (2015). A kinetic study of the CH_2_OO Criegee intermediate self-reaction, reaction with SO2 and unimolecular reaction using cavity ring-down spectroscopy. Phys. Chem. Chem. Phys..

[49] Newland MJ (2015). Kinetics of stabilised Criegee intermediates derived from alkene ozonolysis: reactions with SO_2_, H_2_O and decomposition under boundary layer conditions. Phys. Chem. Chem. Phys..

[50] Kidwell NM, Li H, Wang X, Bowman JM, Lester MI (2016). Unimolecular dissociation dynamics of vibrationally activated CH_3_CHOO Criegee intermediates to OH radical products. Nat. Chem..

[51] Green AM, Barber VP, Fang Y, Klippenstein SJ, Lester MI (2017). Selective deuteration illuminates the importance of tunneling in the unimolecular decay of Criegee intermediates to hydroxyl radical products. Proc. Natl. Acad. Sci. USA.

[52] Drozd GT, Kurtén T, Donahue NM, Lester MI (2017). Unimolecular decay of the dimethyl-substituted Criegee intermediate in alkene ozonolysis: decay time scales and the importance of tunneling. J. Phys. Chem. A.

[53] Lester MI, Klippenstein SJ (2018). Unimolecular decay of Criegee intermediates to OH radical products: prompt and thermal decay processes. Acc. Chem. Res..

[54] Bianchi F (2016). New particle formation in the free troposphere: a question of chemistry and timing. Science.

[55] Tröstl J (2016). The role of low-volatility organic compounds in initial particle growth in the atmosphere. Nature.

[56] Khan MAH, Percival CJ, Caravan RL, Taatjes CA, Shallcross DE (2018). Criegee intermediates and their impacts on the troposphere. Environ. Sci. Process. Impacts.

[57] Rissanen MP (2014). The formation of highly oxidized multifunctional products in the ozonolysis of cyclohexene. J. Am. Chem. Soc..

[58] Jokinen T (2014). Rapid autoxidation forms highly oxidized RO_2_ radicals in the atmosphere. Angew. Chem. Int. Ed..

[59] Chiappini L (2019). Secondary organic aerosol formation from aromatic alkene ozonolysis: influence of the precursor structure on yield, chemical composition, and mechanism. J. Phys. Chem. A.

[60] Long B, Cheng JR, Tan Xf, Zhang Wj (2009). Theoretical study on the detailed reaction mechanisms of carbonyl oxide with formic acid. J. Mol. Struct. THEOCHEM.

[61] Welz O (2014). Rate coefficients of C1 and C2 Criegee intermediate reactions with formic and acetic acid near the collision limit: direct kinetics measurements and atmospheric implications. Angew. Chem. Int. Ed..

[62] Lynch BJ, Zhao Y, Truhlar DG (2003). Effectiveness of diffuse basis functions for calculating relative energies by density functional theory. J. Phys. Chem. A.

[63] Bao JL, Meana-Pañeda R, Truhlar DG (2015). Multi-path variational transition state theory for chiral molecules: the site-dependent kinetics for abstraction of hydrogen from 2-butanol by hydroperoxyl radical, analysis of hydrogen bonding in the transition state, and dramatic temperature dependence of the activation energy. Chem. Sci..

[64] Garrett BC, Truhlar DG (1979). Criterion of minimum state density in the transition state theory of bimolecular reactions. J. Chem. Phys..

[65] Bao JL, Truhlar DG (2017). Variational transition state theory: theoretical framework and recent developments. Chem. Soc. Rev..

[66] Liu YP (1993). Molecular modeling of the kinetic isotope effect for the [1,5]-sigmatropic rearrangement of *cis*-1,3-pentadiene. J. Am. Chem. Soc..

[67] Alecu IM, Zheng J, Zhao Y, Truhlar DG (2010). Computational thermochemistry: scale factor databases and scale factors for vibrational frequencies obtained from electronic model chemistries. J. Chem. Theory Comput..

[68] Klippenstein SJ, Pande V, Truhlar DG (2014). Chemical kinetics and mechanisms of complex systems: a perspective on recent theoretical advances. J. Am. Chem. Soc..

[69] Long B, Bao JL, Truhlar DG (2019). Kinetics of the strongly correlated CH_3_O + O_2_ reaction: the importance of quadruple excitations in atmospheric and combustion chemistry. J. Am. Chem. Soc..

[70] Zheng J, Zhao Y, Truhlar DG (2009). The DBH24/08 database and its use to assess electronic structure model chemistries for chemical reaction barrier heights. J. Chem. Theory Comput..

