# Supplementary material for: Rapid unimolecular reaction of stabilized Criegee intermediates and implications for atmospheric chemistry
Source: Nat Commun. 2019 May 1;10:2003. doi: 10.1038/s41467-019-09948-7 (PMC6494847; doi:10.1038/s41467-019-09948-7)
Supplement: Supplementary file 1 — Supplementary Information [file 41467_2019_9948_MOESM1_ESM.pdf]

SUPPLEMENTARY INFORMATION

Rapid unimolecular reaction of Criegee intermediates and implications for atmospheric chemistry

Supplementary Note 1
Systematic nomenclature

syn-$C_5H_8O_3$: (2*Z*)-(5-oxopentylidene)dioxidan-2-ium-1-ide

anti-$C_5H_8O_3$: (2*E*)-(5-oxopentylidene)dioxidan-2-ium-1-ide

syn-$C_6H_{10}O_3$: (2*Z*)-(6-oxohexylidene)dioxidan-2-ium-1-ide

anti-$C_6H_{10}O_3$: (2*E*)-(6-oxohexylidene)dioxidan-2-ium-1-ide

product of Z-5rc: (1*R*, 5*S*)-6,7,8-trioxabicyclo[3.2.1]octane

product of E-5rc: (1*R*, 5*R*)-6,7,8-trioxabicyclo[3.2.1]octane

product of Z-6rc: (1*R*, 6*S*)-7,8,9-trioxabicyclo[4.2.1]nonane

product of E-6rc: (1*R*, 6*R*)-7,8,9-trioxabicyclo[4.2.1]nonane

product of Z-5hs: (4*E*)-5-hydroperoxypent-4-enal

product of E-5c: 4-(dioxiran-3-yl)butanal

product of Z-6hs: (5*E*)-6-hydroperoxyhex-5-enal

product of E-6c: 5-(dioxiran-3-yl)pentanal

Supplementary Note 2
The multiple conformational structures of (2Z)-(5-oxopentylidene)dioxidan-2-ium-1-ide (*syn*-$C_5H_8O_3$), (2E)-(5-oxopentylidene)dioxidan-2-ium-1-ide (*anti*-$C_5H_8O_3$), and their corresponding transition states

Our general procedure is to rotate all rotatable single bonds by 0, +120, and -120 degrees to generate initial guess for optimizing structures. These optimizations then yield a set of disguisable structures that are used in the MS-T calculations.

There are four torsions in (2Z)-(5-oxopentylidene)dioxidan-2-ium-1-ide (*syn*-$C_5H_8O_3$)

and (2E)-(5-oxopentylidene)dioxidan-2-ium-1-ide (*anti*-$C_5H_8O_3$). They are rotated by 0, 120, and −120 degrees to generate 81 initial conformational structures of each molecule. These 81 initial structures are further optimized by using MN15-L/MGS. This procedure yields 35 and 33 distinguishable structures for *syn*-$C_5H_8O_3$ and *anti*-$C_5H_8O_3$, respectively. The lowest structure of *syn*-$C_5H_8O_3$ is shown in Figures 1 and 2.

For the Z-5hs reaction, because of the envelope-type conformation of the five-member ring in the transition state, there are two classes of the transition state structures: Z-5hs-TS1, in which the substituent –CH_2CH_2CHO is in an equatorial position, and Z-5hs-TS2, in which the substituent –CH_2CH_2CHO is in an axial position. These two classes of the transition state structures are not interconvertable via internal rotation. (Note that the C1-C2 bond in the five-member ring is not rotatable.) Thus, there are only three torsions in each of Z-5hs-TS1 and Z-5hs-TS2, respectively. Optimization yielded 20 and 23 distinguishable structures for Z-5hs-TS1 and Z-5hs-TS2, respectively. The lowest structures are named Z-5hs-TS1 and Z-5hs-TS2 and are shown in Figures 1 and Supplementary Figure 1.

To combine Z-5hs-TS1 and Z-5hs-TS2 into a single MS-T calculation for the transition state and thus yield a single MS-VTST rate constant, the following procedure is used:

(1) We carry out separate conformational searches (rotating all the C-C torsional bonds except for C1-C2 bond) and MS-T calculations for Z-5hs-TS1 and Z-5hs-TS2, and thus we obtain two independent MS-T partition functions, one for Z-5hs-TS1 (denoted as $Q^{\text{MS-T}}$(Z-5hs-TS1)) and one for Z-5hs-TS2 (denoted as $Q^{\text{MS-T}}$(Z-5hs-TS1)).

(2) We name the lowest-energy conformers of Z-5hs-TS1 and Z-5hs-TS2 as GM-Z-5hs-TS1 and GM- Z-5hs-TS2, respectively, where GM denotes global minimum, and we define the potential energy difference (the potential energy for nuclear motion is the Born-Oppenheimer electronic energy including nuclear repulsion):

$$\Delta E = E(\text{GM-Z-5hs-TS1}) - E(\text{GM- Z-5hs-TS2}) \quad (1)$$

(3) If $\Delta E < 0$, as it is in the present case, then the combined MS-T partition function is

$$Q^{\text{MS-T}} = Q^{\text{MS-T}}(\text{Z-5hs-TS1}) + Q^{\text{MS-T}}(\text{Z-5hs-TS2})\cdot\exp(\Delta E/RT) \quad (2)$$

If ΔE were positive, then the combined MS-T partition function would be

$$Q^{\text{MS-T}} = Q^{\text{MS-T}}(\text{Z-5hs-TS1})\cdot\exp(-\Delta E/RT) + Q^{\text{MS-T}}(\text{Z-5hs-TS2}) \quad (3)$$

In these formulas, ΔE is in kcal/mol, and R = 0.0019872036 kcal mol^{-1} K^{-1}. Note the we define partition functions with the zero of energy equal to the equilibrium value of the potential energy – not with respect to the zero-point level.

The same treatment is also used for computing the combined MS-T partition function for reaction Z-6hs.

There are four torsions in E-5c-TS, we use the same procedure to find the distinguishable structures, and we find 59 distinguishable structures for E-5c-TS.

Supplementary Note 3
Check on conformational search

For *syn*-$C_5H_8O_3$, we also did additional calculations to show whether it is sufficient to use structures with rotatable bonds rotated by 0, +120, and -120 degrees to generate initial guesses for optimizing structures. To check this, the four torsions of *syn*-$C_5H_8O_3$ are rotated by 0, 90, 180, and 270 degrees to generate 256 initial conformational structures. This yielded 42 distinguishable structures, 34 of which are the same as the 35 found with 0, +120, and -120 degrees, and 8 of which are new. Then, we used the 42 distinguishable structures to calculate multistructural torsional anharmonicity factor. The calculated results show that the new structures did not have much of an effect on the multistructural torsional anharmonicity factor. With this validation in hand, we use structures obtained with the 0, 120, −120 protocol for calculating all thermodynamic data and rate constants.

Supplementary Note 4
The multiple conformational structures of (2Z)-(6-oxohexylidene)dioxidan-2-ium-1-ide (*syn*-$C_6H_{10}O_3$), (2E)- (6-oxohexylidene)dioxidan-2-ium-1-ide (*anti*-$C_6H_{10}O_3$), and their corresponding transition states:

There are five torsions in (2Z)-(6-oxohexylidene)dioxidan-2-ium-1-ide (*syn*-$C_6H_{10}O_3$), (2E)- (6-oxohexylidene)dioxidan-2-ium-1-ide (*anti*-$C_6H_{10}O_3$), and E-6c-TS. We find 112, 83, and 182 distinguishable structures for *syn*-$C_6H_{10}O_3$, *anti*-$C_6H_{10}O_3$, and E-6c-TS, respectively.

There are four torsions in E-6hs-TS1 and E-6hs-TS2. We find 59 and 59 distinguishable structures for E-6hs-TS1 and E-6hs-TS2. respectively.

Supplementary Note 5
Computational codes

All the electronic structural calculations were performed using *Gaussian 09*,[1] *MN-GFM*,[2] and *Molpro* 2015,[3] and rate constants were calculated using *Polyrat*e*17*-C[4] and *Gaussrate17*-B,[5] and *MSTor*.[6]

Supplementary Table 1. The CCSDT(Q)-CCSD(T) component for Z-5rc-TS.[a] (In kcal/mol)

Quantity	Structure	Value (kcal/mol)
E(CCSDT(Q)/VDZ(d) – *E*(CCSD(T))/VDZ(d)	Z-5rc-TS	0.07

[a] The calculations are done based on the MN15-L/MG3S optimized geometries. The purpose of the comparison of single- point energies by CCSDT(Q)/VDZ(d) and CCSD(T)/VDZ(d) is to show whether post-CCSD(T) calculations are necessary for obtaining a quantitative barrier heights for Z-5rc-TS.

Supplementary Table 2. The unsigned errors (kcal/mol) of the unimolecular reactions of *syn*-$C_5H_8O_3$, *anti*-$C_5H_8O_3$, *syn*-$C_6H_{10}O_3$, and *anti*-$C_6H_{10}O_3$

Method	UE				
	Z-5rc-TS	Z-5hs-TS1	Z-5hs-TS2	E-5rc-TS	E-5c-TS
MN15-L/MG3S[a]	0.36	1.73	0.39	0.94	0.98
MN15-L/maug-cc-pVTZ[a]	0.14	0.94	0.18	1.30	0.63
	Z-6rc-TS	Z-6hs-TS1	Z-6hs-TS2	E-6rc-TS	U6c-TS
MN15-L/MG3S[a]	0.68	0.41	1.02	0.59	0.37
MN15-L/maug-cc-pVTZ[a]	0.67	0.10	0.41	0.22	0.54

[a]The benchmark results (against which errors are calculated) are based on WMS.

Supplementary Table 3. The calculated unimolecular rate constants (s^{-1}) of *syn*-$C_5H_8O_3$, *anti*-$C_5H_8O_3$, *syn*-$C_6H_{10}O_3$, and *anti*-$C_6H_{10}O_3$.

T	Z-5rc	Z-5hs	E-5rc	E-5c	Z-6rc	Z-6hs	E-6rc	E-6c
190	1.34E+09	3.18E+00	3.09E-06	2.57E-04	2.17E+08	5.77E+00	7.81E+05	1.09E-04
200	1.75E+09	5.19E+00	1.84E-05	1.62E-03	2.39E+08	9.01E+00	1.40E+06	7.38E-04
210	2.22E+09	9.11E+00	9.14E-05	8.65E-03	2.59E+08	1.42E+01	2.33E+06	4.14E-03
220	2.74E+09	1.66E+01	3.89E-04	3.98E-02	2.77E+08	2.23E+01	3.65E+06	1.97E-02
230	3.30E+09	3.07E+01	1.45E-03	1.61E-01	2.92E+08	3.51E+01	5.41E+06	8.13E-02
240	3.89E+09	5.69E+01	4.78E-03	5.84E-01	3.04E+08	5.54E+01	7.61E+06	2.95E-01
250	4.50E+09	1.05E+02	1.42E-02	1.91E+00	3.13E+08	8.74E+01	1.02E+07	9.54E-01
260	5.11E+09	1.91E+02	3.88E-02	5.73E+00	3.20E+08	1.38E+02	1.31E+07	2.79E+00
270	5.70E+09	3.46E+02	9.74E-02	1.59E+01	3.24E+08	2.18E+02	1.61E+07	7.43E+00
280	6.26E+09	6.17E+02	2.28E-01	4.11E+01	3.25E+08	3.44E+02	1.92E+07	1.83E+01
290	6.77E+09	1.09E+03	5.00E-01	9.96E+01	3.25E+08	5.44E+02	2.22E+07	4.21E+01
298	7.14E+09	1.69E+03	8.98E-01	1.94E+02	3.24E+08	7.85E+02	2.44E+07	7.84E+01
300	7.22E+09	1.89E+03	1.03E+00	2.28E+02	3.23E+08	8.60E+02	2.50E+07	9.11E+01
310	7.62E+09	3.24E+03	2.04E+00	4.96E+02	3.20E+08	1.35E+03	2.75E+07	1.87E+02
320	7.93E+09	5.47E+03	3.82E+00	1.03E+03	3.16E+08	2.13E+03	2.97E+07	3.68E+02
330	8.17E+09	9.08E+03	6.88E+00	2.05E+03	3.11E+08	3.33E+03	3.16E+07	6.96E+02
340	8.34E+09	1.48E+04	1.19E+01	3.91E+03	3.04E+08	5.17E+03	3.32E+07	1.27E+03
350	8.44E+09	2.39E+04	2.00E+01	7.21E+03	2.98E+08	7.98E+03	3.45E+07	2.26E+03

Supplementary Table 4. The fitted parameters for the unimolecular reactions of *syn*-$C_5H_8O_3$, *anti*-$C_5H_8O_3$, *syn*-$C_6H_{10}O_3$, and *anti*-$C_6H_{10}O_3$

parameter	Z-5rc	Z-5hs	E-5rc	E-5c	Z-6rc	Z-5hs	E-6rc	E-6c
ln *A*	-18.22494	-0.42119	20.97245	23.78909	17.1079	-8.81696	10.49239	27.64743
n	19.34461	24.66698	-1.60892	3.95992	-0.37931	28.32113	-0.24483	-1.95009
T_0 (K)	416.73522	15.39952	40.9312	50.05029	-138.36731	105.05624	-160.63106	22.68511
E (kcal/mol)	-17.60152	-3.8069	11.0785	9.96264	-3.02182	-3.50526	-10.4947	12.81003

Supplementary Table 5. The calculated activation energies (kcal/mol) for the unimolecular reactions of *syn*-$C_5H_8O_3$, *anti*-$C_5H_8O_3$, *syn*-$C_6H_{10}O_3$, and *anti*-$C_6H_{10}O_3$.

T(K)	Z-5rc	Z-5hs	E-5rc	E-5c	Z-6rc	Z-6hs	E-6rc	E-6c
190	1.99	4.27	13.51	13.88	0.75	3.18	4.42	14.59
200	1.98	4.78	13.42	13.95	0.70	3.56	4.36	14.46
210	1.96	5.29	13.34	14.00	0.64	3.95	4.22	14.34
220	1.92	5.8	13.25	14.05	0.57	4.37	4.04	14.22
230	1.86	6.31	13.16	14.09	0.49	4.79	3.82	14.11
240	1.78	6.81	13.07	14.13	0.40	5.24	3.58	14.00
250	1.69	7.31	12.98	14.17	0.31	5.69	3.33	13.90
260	1.58	7.82	12.89	14.20	0.22	6.16	3.06	13.80
270	1.46	8.32	12.8	14.24	0.13	6.64	2.79	13.70
280	1.33	8.82	12.72	14.27	0.04	7.12	2.51	13.61
290	1.19	9.32	12.63	14.3	-0.05	7.62	2.23	13.51
298	1.07	9.72	12.56	14.33	-0.12	8.02	2.01	13.44
300	1.04	9.82	12.55	14.34	-0.14	8.12	1.95	13.43
310	0.88	10.32	12.47	14.37	-0.23	8.62	1.68	13.34
320	0.72	10.82	12.39	14.41	-0.31	9.13	1.4	13.26
330	0.56	11.32	12.31	14.44	-0.4	9.65	1.13	13.18
340	0.39	11.82	12.23	14.48	-0.48	10.17	0.87	13.1
350	0.23	12.31	12.15	14.52	-0.56	10.7	0.61	13.02

Supplementary Table 6. Multistructural torsional anharmonicity factor ($F_{\mathrm{act}}^{\mathrm{MS-T}}$)[a]

T(K)	Z-5rc	Z-5hs-TS	E-5rc	E-5c	Z-6rc	Z-6hs-TS	E-6rc	E-6c
190	0.65	1.37	0.143	0.27	0.052	0.55	0.74	1.28
200	0.65	1.40	0.137	0.27	0.050	0.58	0.72	1.26
210	0.64	1.43	0.131	0.28	0.048	0.62	0.69	1.23
220	0.62	1.46	0.124	0.28	0.045	0.65	0.66	1.20
230	0.61	1.49	0.118	0.29	0.042	0.67	0.62	1.15
240	0.60	1.51	0.112	0.29	0.039	0.69	0.58	1.09
250	0.58	1.53	0.106	0.30	0.037	0.71	0.53	1.03
260	0.56	1.55	0.101	0.30	0.034	0.73	0.48	0.96
270	0.54	1.57	0.095	0.30	0.032	0.74	0.43	0.89
280	0.51	1.57	0.090	0.31	0.029	0.75	0.38	0.82
290	0.49	1.57	0.085	0.31	0.027	0.76	0.33	0.75
298	0.47	1.57	0.081	0.32	0.025	0.76	0.29	0.70
300	0.47	1.57	0.080	0.32	0.025	0.76	0.29	0.69
310	0.44	1.56	0.076	0.32	0.023	0.76	0.25	0.63
320	0.41	1.54	0.071	0.32	0.021	0.77	0.21	0.58
330	0.39	1.52	0.067	0.33	0.020	0.77	0.18	0.54
340	0.36	1.49	0.064	0.33	0.018	0.77	0.16	0.51
350	0.34	1.45	0.060	0.34	0.017	0.77	0.14	0.48

[a] $F_{\mathrm{act}}^{\mathrm{MS-T}} = \frac{F_{\mathrm{TS}}^{\mathrm{MS-T}}}{F_{\mathrm{Reactant}}^{\mathrm{MS-T}}}$, where $F_{\mathrm{Reactant}}^{\mathrm{MS-T}}$ and $F_{\mathrm{TS}}^{\mathrm{MS-T}}$ are calculated using the multistructural method with torsional anharmonicity based on a coupled torsional potential by MN15-L/MG3S. In all cases, F is the ratio of the multiple-structure anharmonic MS-T partition function $Q^{\mathrm{MS-T}}$ to the single-structure quasiharmonic partition function. A quasiharmonic partition function is calculated using the harmonic oscillator formulas but with frequencies scaled to account for anharmonicity and for systematic errors in the electronic structure method being employed. In the present work the scale factors were computed in our general[7] way. The same scale factor is used in the MS-T calculation and in the quasiharmonic calculation. Therefore, F accounts for the effect of multiple structures and torsional potential anharmonicity, including torsion–rotation coupling. For short, we label these combined effects as the multistructural anharmonic effect. Because $F_{\mathrm{act}}^{\mathrm{MS-T}}$ includes both the transition state multistructural anharmonic factor and the reactant multistructural anharmonic factor, it provides the multistructural anharmonic effect on the reaction rate.

Supplementary Table 7. The calculated atmospheric lifetimes (s) for the unimolecular reactions of *syn*-$C_5H_8O_3$, *anti*-$C_5H_8O_3$, *syn*-$C_6H_{10}O_3$, and *anti*-$C_6H_{10}O_3$

T	Z-5rc	Z-5hs	E-5rc	E-5c	Z-6rc	Z-5hs	E-6rc	E-6c
190	7.46E-10	3.14E-01	3.24E+05	3.90E+03	4.60E-09	1.73E-01	1.28E-06	9.14E+03
200	5.71E-10	1.93E-01	5.44E+04	6.17E+02	4.18E-09	1.11E-01	7.16E-07	1.36E+03
210	4.50E-10	1.10E-01	1.09E+04	1.16E+02	3.86E-09	7.06E-02	4.29E-07	2.42E+02
220	3.65E-10	6.02E-02	2.57E+03	2.51E+01	3.61E-09	4.49E-02	2.74E-07	5.07E+01
230	3.03E-10	3.25E-02	6.92E+02	6.20E+00	3.43E-09	2.85E-02	1.85E-07	1.23E+01
240	2.57E-10	1.76E-02	2.09E+02	1.71E+00	3.29E-09	1.81E-02	1.31E-07	3.39E+00
250	2.22E-10	9.54E-03	7.02E+01	5.23E-01	3.19E-09	1.14E-02	9.81E-08	1.05E+00
260	1.96E-10	5.22E-03	2.58E+01	1.74E-01	3.13E-09	7.24E-03	7.65E-08	3.59E-01
270	1.75E-10	2.89E-03	1.03E+01	6.29E-02	3.09E-09	4.58E-03	6.20E-08	1.35E-01
280	1.60E-10	1.62E-03	4.39E+00	2.44E-02	3.07E-09	2.90E-03	5.21E-08	5.46E-02
290	1.48E-10	9.20E-04	2.00E+00	1.00E-02	3.07E-09	1.83E-03	4.51E-08	2.38E-02
298	1.40E-10	5.90E-04	1.11E+00	5.16E-03	3.09E-09	1.27E-03	4.09E-08	1.28E-02
300	1.38E-10	5.29E-04	9.67E-01	4.39E-03	3.09E-09	1.16E-03	4.00E-08	1.10E-02
310	1.31E-10	3.09E-04	4.91E-01	2.02E-03	3.12E-09	7.36E-04	3.64E-08	5.34E-03
320	1.26E-10	1.83E-04	2.62E-01	9.72E-04	3.17E-09	4.69E-04	3.37E-08	2.72E-03
330	1.22E-10	1.10E-04	1.45E-01	4.89E-04	3.22E-09	3.00E-04	3.16E-08	1.44E-03
340	1.20E-10	6.74E-05	8.38E-02	2.56E-04	3.28E-09	1.93E-04	3.01E-08	7.86E-04
350	1.19E-10	4.19E-05	5.00E-02	1.39E-04	3.36E-09	1.25E-04	2.90E-08	4.43E-04

Supplementary Table 8. Absolute energies (in hartrees) of optimized structures by CCSD(T)-F12a/jun-cc-pVTZ

Compound	Absolute energy
syn-$C_5H_8O_3$	-420.38106330
Z-5rc-TS	-420.37828975
Z-5rc-TS1	-420.37238485
Z-5rc-P	-420.43918135
Z-5hs-TS1	-420.34917171
Z-5hs-P1	-420.40851196
Z-5hs-TS2	-420.34284366
anti-$C_5H_8O_3$	-420.37103815
E-5rc-TS	-420.35042814
E-5rc-P	-420.43918138
E-5c-TS	-420.34789672
E-5c-P	-420.41246623
syn-$C_6H_{10}O_3$	-459.63251358
Z-6rc-TS	-459.63096400
Z-6rc-TS1	-459.62808727
Z-6rc-TS2	-459.62516971
Z-6rc-TS3	-459.61920114
Z-6rc-P	-459.68917854
Z-6hs-TS1	-459.60237669
Z-6hs-P1	-459.66094675
Z-6hs-TS2	-459.59907293
anti-$C_6H_{10}O_3$	-459.62933416
E-6rc-TS	-459.62293869
E-6rc-TS1	-459.61744637
E-6rc-P	-459.70007661
E-6c-TS	-459.60487618
E-6c-P	-459.66835416
TSR1	-420.31177454
TSR2	-459.56963511

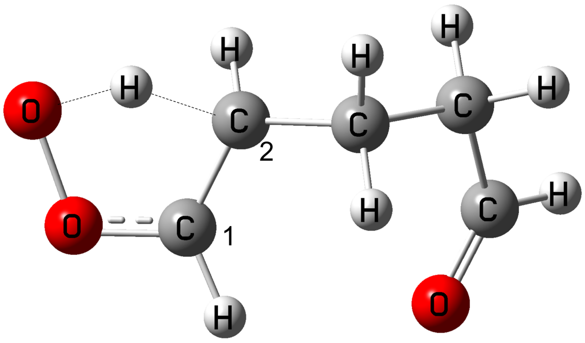

Z-5hs-TS1

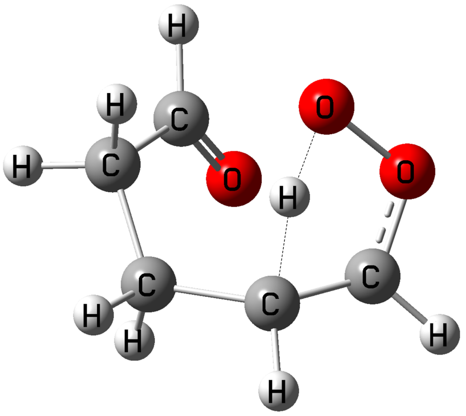

Z-5hs-TS2

Supplementary Figure 1. The optimized transition states for the 1,4-hydrogen shift of *syn*-$C_6H_{10}O_3$ by MN15-L/MG3S. The 35 distinguishable structures for *syn*-C_5H_8O involve some structures differing only by rotation of the C1-C2 bond of the *syn*-$C_5H_8O_3$ reactant with the other carbon-carbon bonds fixed. In the transition states, the C1-C2 bond cannot be rotated and other carbon-carbon bonds are rotated to produce the two lowest-energy transition states Z-5hs-TS1 and Z-5hs-TS2.

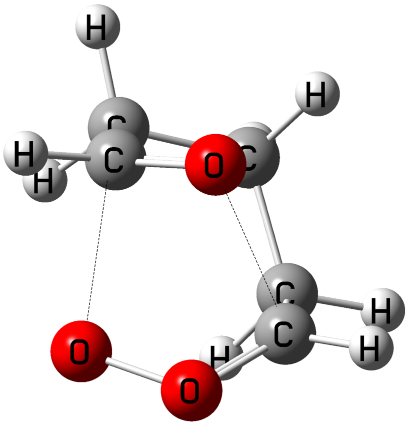

Z-5rc-TS

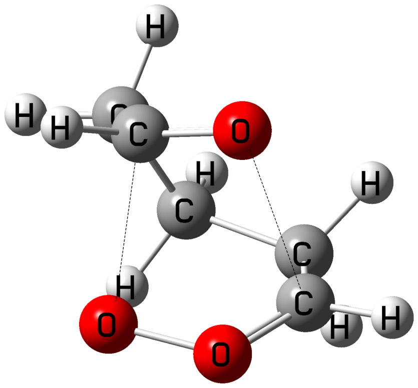

Z-5rc-TS1

Supplementary Figure 2. Two different transition state structures for SOZ formation in *syn*-$C_5H_8O_3$ calculated by MN15-L/MG3S. The calculated enthalpies of activation at 0 K are 2.27 and 5.64 kcal/mol for Z-5rc-TS and Z-5rc-TS1, respectively by CCSD(T)-F12a/jun-cc-pVTZ//MN15-L/MG3S.

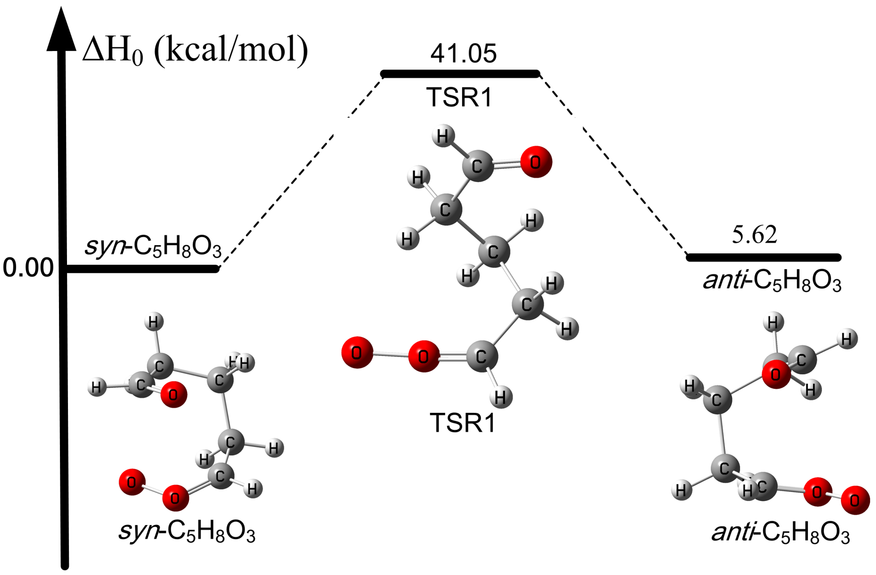

Supplementary Figure 3. The calculated enthalpy profile of *syn*-$C_5H_8O_3$ transferred to *anti*-$C_5H_8O_3$ by CCSD(T)-F12a/jun-cc-pVTZ//MN15-L/MG3S.

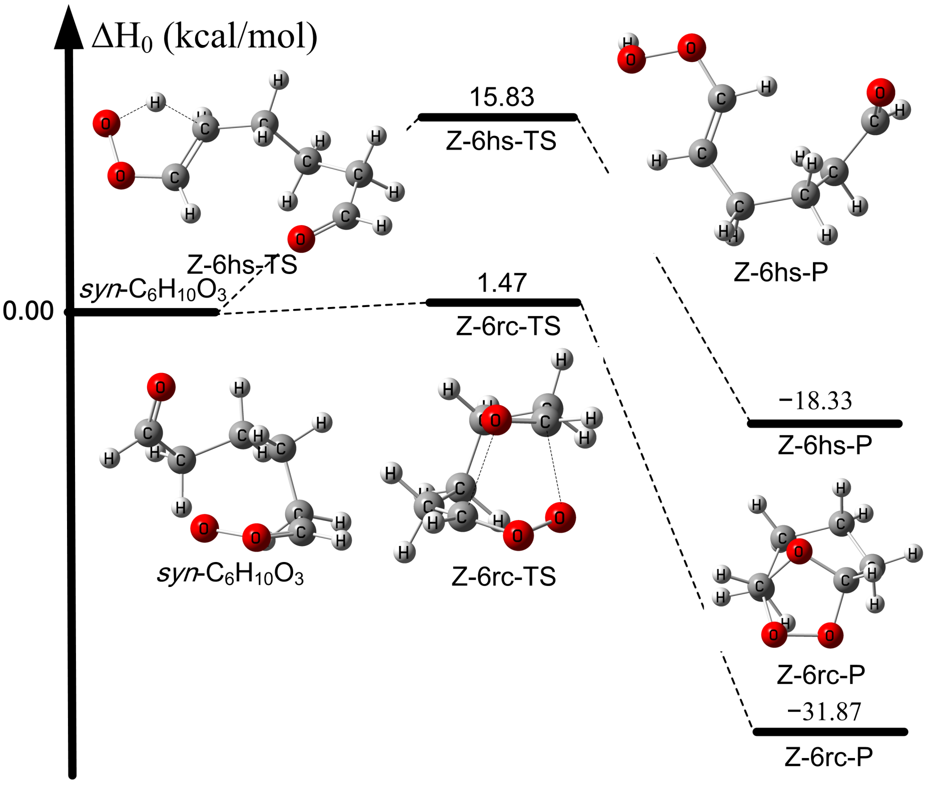

Supplementary Figure 4. The calculated enthalpy profile of *syn*-$C_6H_{10}O_3$ by CCSD(T)-F12a/jun-cc-pVTZ//MN15-L/MG3S.

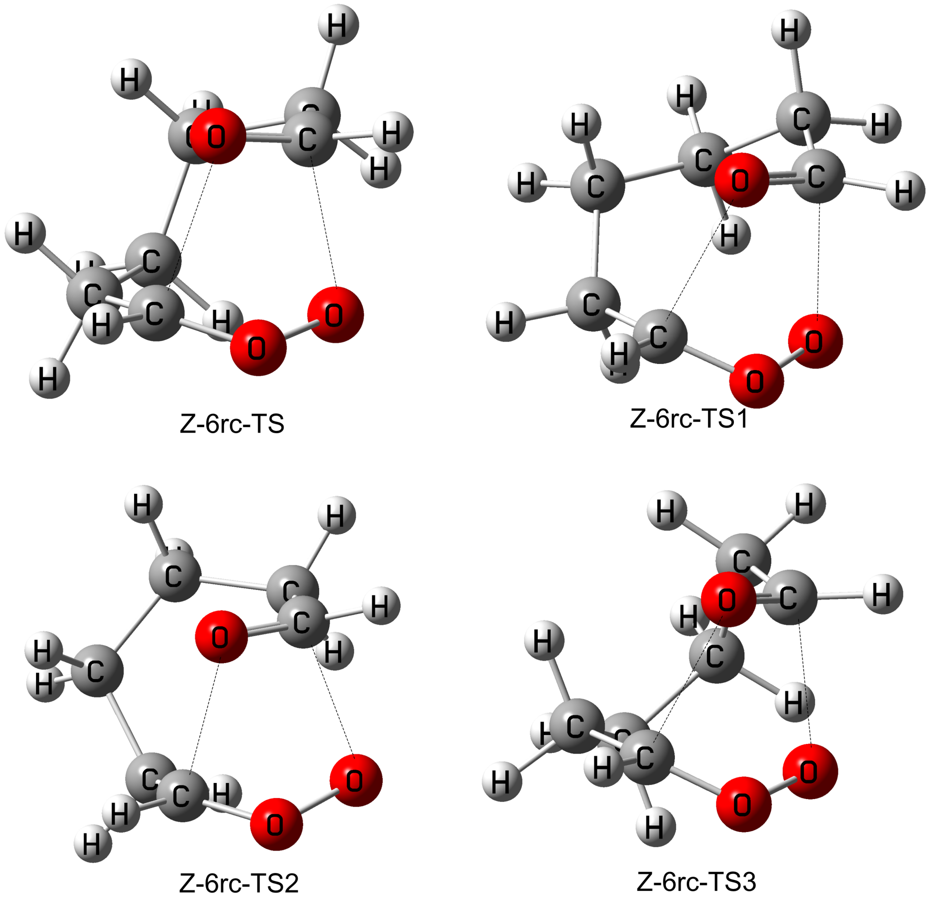

Supplementary Figure 5. Four different optimized transition state structures for SOZ formation in *syn*-$C_6H_{10}O_3$ calculated by MN15-L/MG3S. The calculated enthalpies of activation at 0 K are 1.47, 3.44, 5.36, and 9.15 kcal/mol for Z-6rc-TS, Z-6rc-TS1, Z-6rc-TS2, and Z-6rc-TS3, respectively by CCSD(T)-F12a/jun-cc-pVTZ//MN15-L/MG3S. The previous investigation[8] reported Z-6rc-TS2, not Z-6rc-TS.

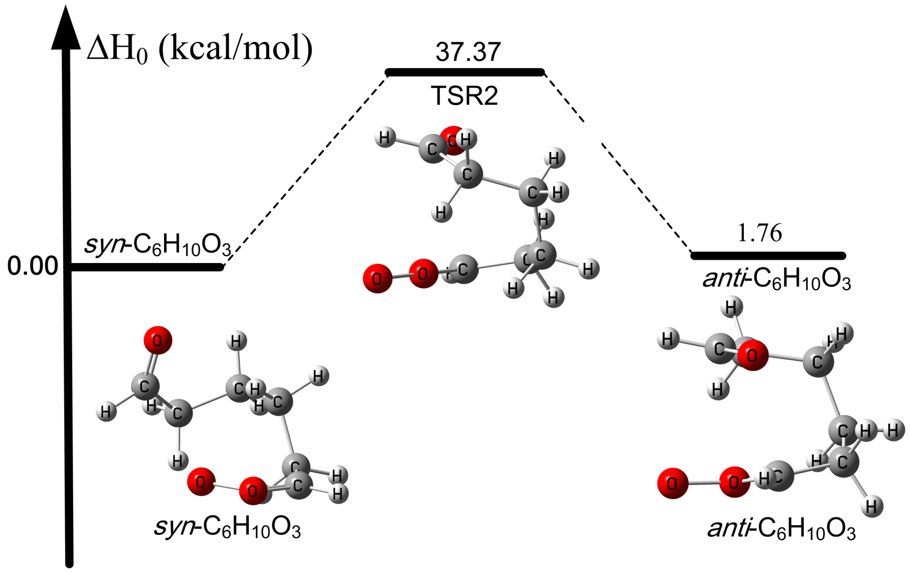

Supplementary Figure 6. The calculated enthalpy profile of *syn*-$C_6H_{10}O_3$ transferred to *anti*-$C_6H_{10}O_3$ by CCSD(T)-F12a/jun-cc-pVTZ//MN15-L/MG3S.

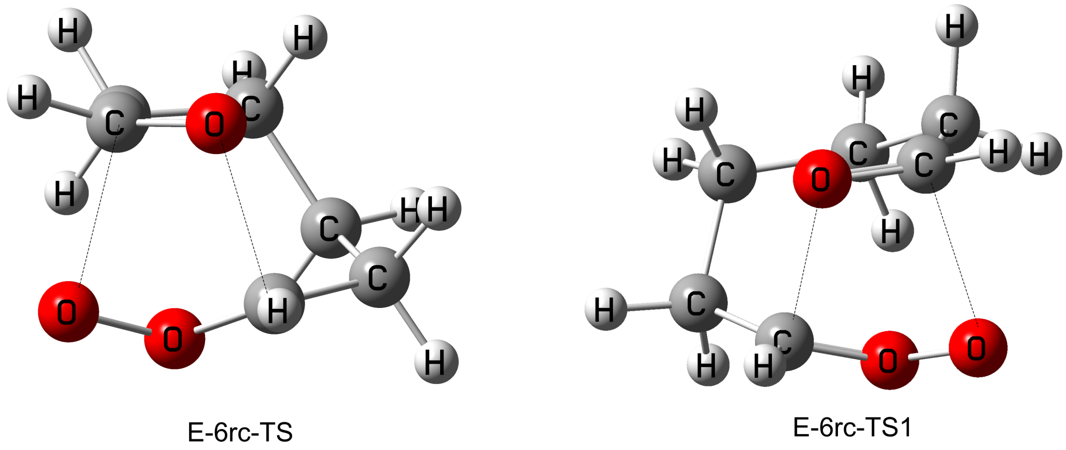

Supplementary Figure 7. The optimized three different transition state structures for SOZ formation in *anti*-$C_6H_{10}O_3$ calculated by MN15-L/MG3S. The calculated enthalpies of activation at 0 K are 4.82, 8.23 kcal/mol for E-6rc-TS, E-6rc-TS1, respectively by CCSD(T)-F12a/jun-cc-pVTZ//MN15-L/MG3S.

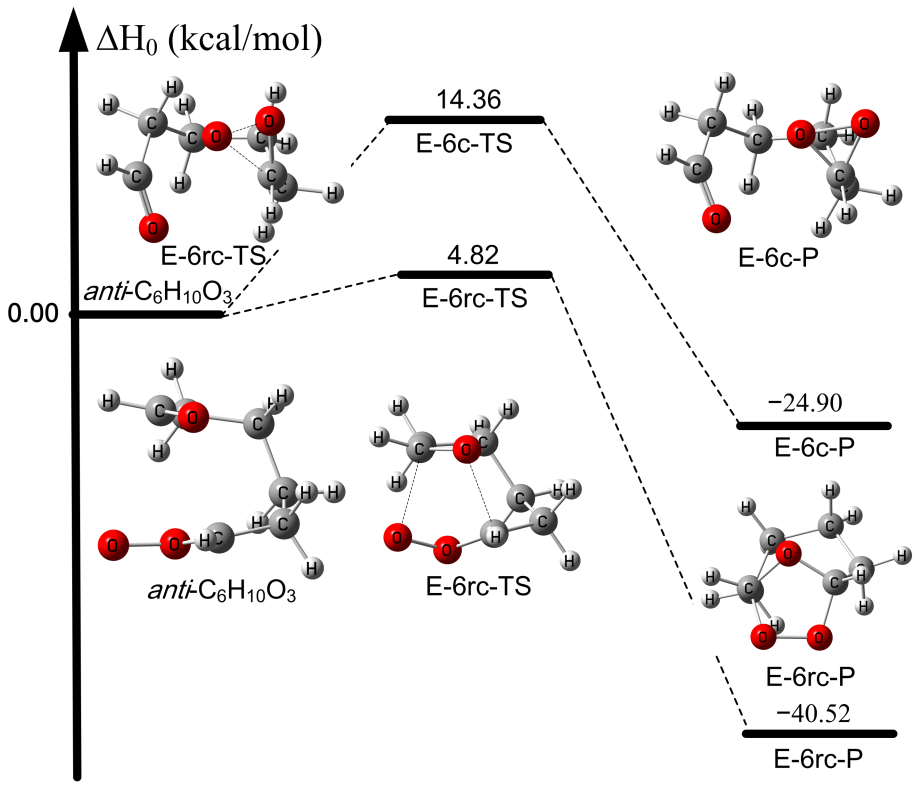

Supplementary Figure 8. The calculated enthalpy profile of *anti*-$C_6H_{10}O_3$ by CCSD(T)-F12a/jun-cc-pVTZ//MN15-L/MG3S.

Supplementary References

[1] Frisch, M. J., Trucks, G. W., Schlegel, H. B., Scuseria, G. E., Robb, M. A., Cheeseman, J. R., Scalmani, G., Barone, V., Mennucci, B., Petersson, G. A., Nakatsuji, H., Caricato, M., Li, X., Hratchian, H. P., Izmaylov, A. F., Bloino, J., Zheng, G., Sonnenberg, J. L., Hada, M., Ehara, M., Toyota, K., Fukuda, R., Hasegawa, J., Ishida, M., Nakajima, T., Honda, Y., Kitao, O., Nakai, H., Vreven, T., Montgomery, J. A. Jr., Peralta, J. E., Ogliaro, F., Bearpark, M., Heyd, J. J., Brothers, E., Kudin, K. N., Staroverov, V. N., Kobayashi, R., Normand, J., Raghavachari, K., Rendell, A., Burant, J. C., Iyengar, S. S., Tomasi, J., Cossi, M., Rega, N., Millam, J. M., Klene, M., Knox, J. E., Cross, J. B., Bakken, V., Adamo, C., Jaramillo, J., Gomperts, R., Stratmann, R. E., Yazyev, O., Austin, A. J., Cammi, R., Pomelli, C., Ochterski, J. W., Martin, R. L., Morokuma, K., Zakrzewski, V. G., Voth, G. A., Salvador, P., Dannenberg, J. J., Dapprich, S., Daniels, A. D., Farkas, O., Foresman, J. B., Ortiz, J. V., Cioslowski, J. & Fox, D. J. Gaussian 09, revision C.01, Gaussian, Inc.: Wallingford, CT, 2010.

[2] Zhao, Y., Peverati, R., Tang, K., Luo, S., Yu, H. S., He, X. & Truhlar, D. G. *MN-GFM 6.7*, Department of Chemistry, University of Minnesota: Minneapolis, MN 55455-0431, 2015.

[3] Werner, H.-J., Knowles, P. J., Knizia, G., Manby, F. R., Schütz, M., Celani, P., Korona, T., Lindh, R., Mitrushenkov, A., Rauhut, G., Shamasundar, K. R., Adler, T. B., Amos, R. D., Bernhardsson, A., Berning, A., Cooper, D. L., Deegan, M. J. O., Dobbyn, A. J., Eckert, F., Goll, E., Hampel, C., Hesselmann, A., Hetzer, G., Hrenar, T., Jansen, G., Köppl, C., Liu, Y., Lloyd, A. W., Mata, R. A., May, A. J., McNicholas, S. J., Meyer, W., Mura, M. E., Nicklass, A., O'Neill, D. P., Palmieri, P., Pflüger, K., Pitzer, R., Reiher, M., Shiozaki, T., Stoll, H., Stone, A. J., Tarroni, R., Thorsteinsson, T., Wang, M. & Wolf, A. *Molpro*, version 2015.1, A Package of Ab Initio Programs, http://www.molpro.net.

[4] Zheng, J., Bao, J. L., Meana-Pañeda, R., Zhang, S., Lynch, B. J., Corchado, J. C., Chuang, Y.-Y., Fast, P. L., Hu, W.-P., Liu, Y.-P., Lynch, G. C., Nguyen, K. A.,Jackels, C. F., Fernandez-Ramos, A., Ellingson, B. A., Melissas, V. S.,Villa, J., Rossi, I., Coitino, L., Pu, J., Albu, T. V., Steckler, R., Garrett, B. C., Issacson A. D., & Truhlar, D. G. POLYRATE17-C, University of Minnesota: Minneapolis, 2018.

[5] Zheng, J., Bao, J. L., Zhang, S., Corchado, J. C., Chuang, Y.-Y., Coitiño, E. L., Ellingson, B. A. & Truhlar, D. G. GAUSSRATE17-B, University of Minnesota: Minneapolis, 2018.

[6] Zheng, J., Mielke, S. L., Bao, J. L., Meana-Pañeda, R., Clarkson, K. L., & Truhlar, D. G., MSTor computer program, version 2017-B, University of Minnesota, Minneapolis, MN, 2017.

[7] "Computational Thermochemistry: Scale Factor Databases and Scale Factors for Vibrational Frequencies Obtained from Electronic Model Chemistries," I. M. Alecu, J. Zheng, Y. Zhao, and D. G. Truhlar, Journal of Chemical Theory and Computation **6**, 2872-2887 (2010).

[8] Chuong, B., Zhang, J. & Donahue, N. M. Cycloalkene Ozonolysis: Collisionally Mediated Mechanistic Branching. *J. Am. Chem. Soc.* **126**, 12363-12373 (2004).
