# Supplementary material for: Rapid unimolecular reaction of stabilized Criegee intermediates and implications for atmospheric chemistry
Source: Nat Commun. 2019 May 1;10:2003. doi: 10.1038/s41467-019-09948-7 (PMC6494847; doi:10.1038/s41467-019-09948-7)
Supplement: Supplementary file 2 — Description of Additional Supplementary Files [file 41467_2019_9948_MOESM2_ESM.pdf]

Description of Additional Supplementary Files

File Name: Supplementary Data 1
Description: Cartesian coordinates (Å) of structures optimized by MN15-L/MG3S
